# From Strengths to Flourishing: A Parallel Mediation Model of Strengths Self-Efficacy and Resilience Among Student Teachers

**DOI:** 10.3390/bs16050628

**Published:** 2026-04-23

**Authors:** Thet Thet Mar, Hijjatul Qamariah, Mária Hercz

**Affiliations:** 1 Doctoral School of Education, Eötvös Loránd University, 1053 Budapest, Hungary; thetthet@student.elte.hu; 2 Department of Educational Studies, Meiktila Education Degree College, Meiktila 05181, Myanmar; 3 Doctoral School of Education, University of Szeged, 6720 Szeged, Hungary; hercz.maria@gmail.com

**Keywords:** character strengths, strengths self-efficacy, resilience, flourishing, student teachers, structural equation modeling

## Abstract

A cross-sectional study was conducted with student teachers from four Education Degree Colleges located in Upper and Lower Myanmar. Drawing on the positive psychology framework, the predictive role of character strengths in flourishing was examined by integrating strengths self-efficacy (SSE) and resilience as parallel mediators. Participants (*n* = 1251, *M*_age_ = 20.84 years, SD = 1.28) were selected using stratified random sampling and completed four validated measures: VIA-72, SSE Scale, Connor–Davidson Resilience Scale 25, and Flourishing Scale. Correlational analyses revealed significant moderate positive associations between study variables. Using structural equation modeling, the results showed a direct predictive effect of character strengths on SSE, resilience, and flourishing. In addition, SSE and resilience partially mediated the relationship between character strengths and flourishing. Importantly, the indirect pathway through resilience was stronger than the SSE, indicating that the ability to adapt to challenges plays an essential role in linking character strengths with the flourishing of student teachers in the Myanmar Teacher Education setting, which practices a competency-based curriculum. Overall, supporting the strengths-based literature, the parallel mediational model of SSE and resilience contributes to a better understanding of how character strengths explain flourishing. The implications for Teacher Education and directions for future research are discussed.

## 1. Introduction

Modern education systems prepare young people for the rapidly evolving labor market, in which increasing societal demands and advancing technology reshape the nature of work. Consequently, the mental health crisis among young people contributes to feelings of inadequacy for the future, leading education policymakers to rethink how they can provide quality education to individuals. The future education system should train students to cultivate a broad range of strengths, allowing them to establish societal and organizational models that reconcile competing beliefs, values, meaning, and purpose in life for individual fulfillment and the development of flourishing (OECD, 2025). In this context, Teacher Education aims to nurture student teachers with the necessary competencies to navigate uncertainties effectively and contribute to flourishing societies. Accordingly, Initial Teacher Education (ITE) plays an essential role not only in providing effective practice and shaping the professional identity of good teachers, but also in supporting student teachers’ well-being (Darling-Hammond, 2017).

In the context of Myanmar Teacher Education, student teachers are trained to meet the essential qualities required by a competency-based curriculum. Education Degree Colleges (EDCs) offer a four-year Bachelor of Education program aligned with the Teacher Competency Standards Framework implemented since 2019 (Lall, 2021). The competency-based curriculum provides opportunities for students to develop theoretical knowledge, pedagogical skills, and practical teaching experience, as well as participation in co-curricular activities, dormitory life, cultural events, and social engagement. While the teaching–learning environment is designed to be active and engaging to enhance teacher quality, these multiple demands may create difficulties for student teachers when it comes to effectively managing and adapting to academic and social responsibilities, particularly during the early years of training. In this regard, adaptability and maintaining a positive attitude, along with awareness of one’s strengths, are crucial to achieving a balanced, meaningful life, which encompasses social, emotional, and psychological well-being within the college environment (Yıldırım & Çelik Tanrıverdi, 2020). However, empirical research on well-being and flourishing within Myanmar Teacher Education remains limited, with most existing work confined to gray literature and little attention given to the role of character strengths (Mar & Hercz, 2024). Therefore, it is important to investigate the key psychological mechanisms that contribute to the flourishing of student teachers in this context.

Conceived in this way, Mar et al. (2025) highlighted that educational environments should prioritize supportive classroom climates that foster the holistic development of individuals, encouraging the use of personal strengths and positivity, which are key to flourishing. Building on the work of Diener et al. (2010), the origins of the flourishing concept can be traced to the humanistic tradition, different approaches to well-being, and positive psychology. A wealth of literature conceptualizes flourishing as a comprehensive framework to understand optimal human functioning. For instance, Huppert and So (2013) define flourishing as a combination of feeling good and functioning effectively, while Keyes (2002) describes flourishing as high levels of emotional, psychological, and social well-being. Concerning the different perspectives of well-being: the hedonic perspective, which focuses on the cognitive and affective parts, where the individuals experience positive and negative affect, life satisfaction, and happiness (Diener, 2000); and the eudaimonic perspective, in which Ryff (2014) explained the six dimensions of psychological well-being (self-acceptance, purpose in life, environmental mastery, positive relationships, personal growth, and autonomy). The influential perspective on flourishing integrates hedonic (Diener, 1984) and eudaimonic well-being traditions (Ryff & Keyes, 1995), as well as strengths-based perspectives from the positive psychological framework, considering the global flourishing dimensions (Zambelli et al., 2025). Therefore, in the current study, flourishing can be defined as a psychological construct that represents the full functioning of a person experiencing a balance between positive and negative affect, and satisfaction, linking high levels of social and psychological well-being.

Research has shown that the cultivation of character strengths serves as a foundational psychological tool to achieve a flourishing life, and strengths-related behavior is a better predictor of flourishing (Duan & Ho, 2018; Wagner et al., 2021). To promote flourishing, the critical role of character strengths is highlighted, introducing a model that underscores how character strengths empower individuals to thrive, build resilience during adversity, and support individual and collective flourishing (Mayerson, 2020). The current study adopts this perspective, viewing character strengths as essential psychological resources that help people respond constructively to challenges, thus enhancing flourishing while maintaining harmonious relationships with others. In order to expand the existing literature, this study integrated Tsai et al.’s (2014) recommendations that emphasize the importance of self-efficacy in translating strengths awareness into strengths-related behavior in everyday life. Strengths self-efficacy (SSE) represents individuals’ confidence to apply their personal strengths within their surroundings. The pivotal role of SSE is essential in fostering individuals to lead a productive life, feel good, and function effectively. Considering the above-mentioned theoretical background and empirical findings, this study examined the mediating roles of SSE and resilience in the relationship between character strengths and the flourishing of student teachers.

### 1.1. A Strengths-Based Perspective on Flourishing

Character strengths are rooted in the principles of positive psychology, different cultural traditions, and religions, enabling individuals to achieve their fullest potential without diminishing others’ benefits (Peterson & Seligman, 2004). The Values in Action (VIA) classifies 24 character strengths, organized under six virtues (Wisdom and knowledge, Courage, Humanity, Justice, Temperance, and Transcendence), that reflect positive personality traits. Individuals express these strengths to varying degrees depending on life demands and circumstances. Reflecting on the past 20 years of the character strengths’ contribution across different disciplines, a recent bibliometric analysis by Feraco and Casali (2025) showed the significant impact of character strengths in well-being research, highlighting the increasing prevalence of cross-cultural research in non-WEIRD (Western, Educated, Industrialized, Rich, and Democratic) contexts. While several measures have been developed and validated to assess character strengths, a growing body of literature has provided empirical evidence of the instability factor structure based on an analysis of the 24 character strengths. The resulting factors deviate from the original VIA model with a varying number of factors (see, e.g., three factors; Duan & Bu, 2017, four factors; McGrath & Walker, 2016, five factors; Azañedo et al., 2021, and six factors; Ng et al., 2017).

In light of these findings, the current study adopts a higher-order four-factor model: (1) intellectual and emotional strengths (creativity, curiosity, love of learning, perspective, judgement, nature of beauty and excellence, bravery and prudence), (2) intrapersonal strengths (self-regulation, honesty, perseverance, spirituality, humility, and hope), (3) interpersonal strengths (kindness, social intelligence, love, humor, gratitude, and zest) and (4) civic strengths (fairness, teamwork, leadership and forgiveness), identified through exploratory and confirmatory factor analyses. This structure provides a parsimonious representation of the strengths relevant to the present Burmese sample. Regardless of the variation in the factor structure, it shares a common goal with flourishing based on Aristotle’s notion of eudaemonia: a good life characterized by virtues and meaningful engagement, where actions are intrinsically fulfilling (Peterson & Seligman, 2004). In addition, a substantial body of literature has documented the link between character strengths and flourishing, including simple correlational studies, mediational analyses, and intervention programs across education, the workplace, and clinical settings. For instance, research has revealed the robust links to flourishing among university students (Charles-Leija et al., 2025), college students (Jafari, 2020), and teachers (Mariana et al., 2023; Yeo, 2011), emphasizing the significance of character strengths in flourishing.

The Harvard Human Flourishing Program has further contributed to educational practices through evidence-informed approaches that foster character development programs to enhance students’ well-being and flourishing (Hinton et al., 2023). Beyond education settings, the contribution of character strengths extends to workplace flourishing. Madhu and Swati (2025) explored how intellectual strengths support organizational happiness and well-being, suggesting practical strategies (e.g., mentorship programmes focusing on character strengths application, feedback loop prioritizing constructive interaction, and interdisciplinary cooperation emphasizing broaden perspectives) for employee engagement and flourishing. Similarly, the adult community sample also showed associations between 24 character strengths and flourishing (Bachman et al., 2024). In clinical contexts, Yan et al. (2020) supported the effectiveness of character strengths-based interventions to help improve the well-being, self-esteem, self-efficacy, and happiness, while reducing depression and mental health problems of individuals with chronic illness.

Given the substantial empirical evidence linking character strengths to flourishing, further exploration in Teacher Education is essential. A recent systematic literature review of character strengths in Teacher Education revealed that intellectual strengths are related to happiness and transcendent strengths are related to life satisfaction among student teachers (Yildiz, 2025). Mar and Hercz (2024) highlighted the practical relevance of 24 character strengths in the teaching–learning environment, emphasizing the professional development of student teachers. However, there is still limited empirical research examining the role of character strengths in student teachers’ flourishing, and more comprehensive quantitative investigations are needed to determine the extent to which character strengths affect flourishing in the Teacher Education setting. To address this gap, the present study aims to investigate how character strengths relate to flourishing, specifically through resilience and strengths self-efficacy.

### 1.2. Resilience as an Adaptive Pathway Linking Character Strengths and Flourishing

Resilience is defined as the comprehensive ability to manage stress and adversity, enabling individuals to thrive despite difficulties (Connor & Davidson, 2003). It functions as a key adaptive mechanism that facilitates coping with stress, promotes healthy behaviors, and supports flourishing (Baek et al., 2010; Cohn et al., 2009). Resilient individuals are better able to navigate and transform challenges while maintaining the pursuit of their goals to foster happiness, well-being, and academic success. Regarding social relationships, they are often able to extend their support to others in need (Karris-Bachik et al., 2021; Kim et al., 2017). Within this framework, character strengths are instrumental in overcoming challenges with positivity and adaptive coping strategies, contributing to flourishing outcomes (Harzer & Ruch, 2015; Niemiec, 2019).

Drawing on Mayerson’s (2020) character strengths response model, these strengths serve as psychological resources that enable individuals to facilitate positive adaptation and maintain a purposeful life. Consequently, resilience can be understood as a key pathway through which character strengths translate into flourishing. Empirical research provides support for this mechanism, demonstrating that character strengths are positively associated with resilience, which in turn contributes to higher levels of well-being and flourishing (De la Fuente et al., 2022; Ekman & Simon-Thomas, 2021; Martínez-Martí & Ruch, 2017; Yildirim & Belen, 2019). Taken together, it is proposed that resilience functions as a significant mediator in the relationship between character strengths and flourishing among student teachers in Myanmar.

### 1.3. Strengths Self-Efficacy (SSE) as a Motivational Mechanism in Linking Character Strengths and Flourishing

In addition to the adaptive role of resilience, self-efficacy plays an important role in the association between character strengths and flourishing. Although existing studies have largely focused on self-efficacy, these insights provide a relevant foundation for understanding strengths self-efficacy (SSE). SSE refers to individuals’ confidence in their ability to leverage their character strengths in daily life (Tsai et al., 2014). Strengths-based research has consistently demonstrated a positive relationship between character strengths and self-efficacy. Intellectual and temperance strengths have emerged as the strongest predictors of self-efficacy beliefs, while specific strengths such as leadership, temperance, and intellectual strengths have been linked to higher self-efficacy among adolescents (Dronnen-Schmidt, 2014; Weber et al., 2013). Similarly, higher-order factors of character strengths, including emotional, intellectual, restraint, and theological strengths, have been shown to correlate positively with self-efficacy (Martínez-Martí & Ruch, 2017).

These findings suggest that character strengths may enhance individuals’ confidence in their capabilities, which is conceptually aligned with the construct of strengths self-efficacy. Extending this line of research, self-efficacy was also identified as a significant mediator in the relationship between character strengths and psychological well-being, suggesting a pattern in which strengths contribute to enhanced psychological well-being through increased efficacy beliefs (Xie et al., 2020). Theoretically, this mechanism is grounded in Bandura’s (1997) self-efficacy theory, which posits that strong self-efficacy beliefs encourage individuals to implement strengths and influence their daily task performance. Within a strengths-based framework, strengths self-efficacy functions as a domain-specific form of self-efficacy that facilitates the effective mobilization of personal strengths, particularly in challenging situations. According to Zhao et al. (2021), the character strengths-based intervention group showed that adolescents who are aware of their character strengths and learn how to use them in daily life build great confidence in their ability to cope, promoting their well-being and reducing anxiety. People with high strengths self-efficacy tend to report greater confidence in applying their strengths, leading to greater well-being and flourishing. Although empirical research explicitly focusing on SSE remains limited, emerging evidence supports its role as a motivational mechanism in activating strengths and promoting positive outcomes (Tsai et al., 2014; Wang & Ding, 2023). Consequently, SSE is conceptualized as an essential pathway in linking character strengths to flourishing.

### 1.4. Gender Differences in Study Variables

A recent meta-analysis encompassing 65 studies has highlighted notable gender differences in character strengths, although these differences exhibit small effect sizes. From a social constructionist perspective, such differences are context-dependent and reflect socially constructed meanings associated with being male and female. This perspective highlights the importance of examining whether the relationships between the study variables are consistent across gender groups (Heintz et al., 2019). Previous research provided evidence of gender’s significant and modest predictive role in resilience, character strengths, flourishing, and strengths self-efficacy (Ekşi et al., 2022; Ruch et al., 2014). However, empirical evidence remains inconclusive: some studies have incorporated gender as a control variable (Casali et al., 2021; Martínez-Martí & Ruch, 2017), whereas others excluded it due to its lack of significant effects (Shuakbayeva et al., 2025; Zhao et al., 2021). Given these inconsistent findings, it is imperative to further investigate the associations between gender and the study variables.

### 1.5. Aim and Hypotheses

**Figure 1:**
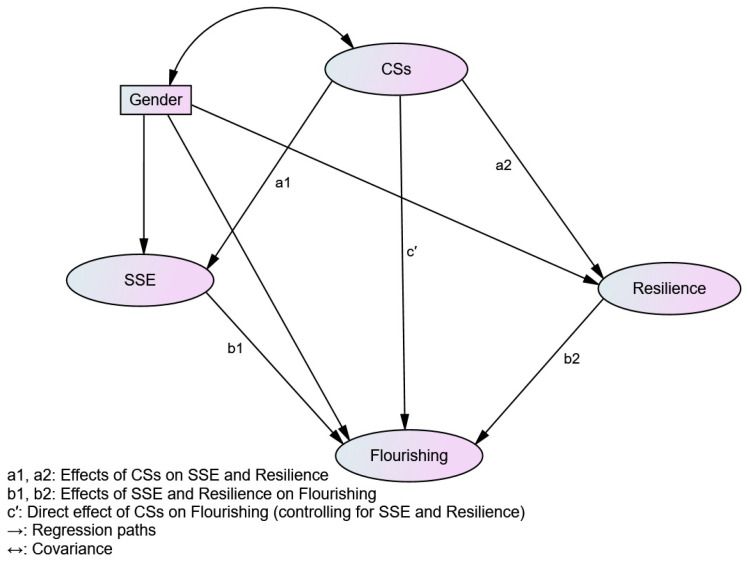
Conceptual model of the parallel mediation effects of strengths self-efficacy (SSE) and resilience in the relationship between character strengths (CSs) and flourishing.

Guided by the theoretical and empirical studies discussed above, SSE and resilience appear to play a significant role in the association between character strengths and flourishing. The mediating roles of resilience and SSE have not yet been documented in a single model for character strengths and flourishing, despite previous studies exploring these constructs separately. To address this gap, the present study aimed to investigate a parallel mediation model in which resilience and strengths self-efficacy jointly explain how character strengths contribute to flourishing (see Figure 1). To the best of our knowledge, this is the first study to examine a parallel mediational framework in the context of Myanmar Teacher Education, thereby providing a more comprehensive understanding of the psychological mechanisms underlying student teachers’ flourishing. Within this framework, character strengths are expected to enhance flourishing directly and indirectly through SSE and resilience. Student teachers with high prevalence of character strengths are more likely to believe in their ability to use these strengths to support flourishing and to adapt effectively to challenges, which in turn promotes their flourishing in the Teacher Education context. In addition, gender was incorporated as a control variable within the structural model to account for its potential impact on the relationships between the study variables. As suggested by previous research, the following hypotheses are proposed:

**H1.**  *Character strengths significantly predict strengths self-efficacy, resilience, and flourishing among student teachers in Myanmar (De la Fuente et al., 2022; Yildirim & Belen, 2019).*

**H2.**  *Strengths self-efficacy and resilience partially mediate the relationship between character strengths and flourishing among student teachers in Myanmar (De la Fuente et al., 2022; Wang & Ding, 2023; Yildirim & Belen, 2019).*

## 2. Methodology

### 2.1. Participants and Procedure

This study employed a cross-sectional, correlational research design using structural equation modeling (SEM) to investigate the parallel mediating roles of resilience and strengths self-efficacy in the relationship between character strengths and flourishing of Myanmar student teachers (Creswell, 2012). A stratified random sampling was employed to obtain comprehensive research data and improve generalizability (Mills & Gay, 2016). Data were collected from four Education Degree Colleges (EDCs) out of twenty-five EDCs located in Upper and Lower Myanmar. Participation was voluntary, and teacher educators from the respective EDCs distributed the secure Qualtrics survey to student teachers after obtaining data collection permission from the Ministry of Education of Myanmar. Data were collected from June to August 2025, and a total of 1251 valid responses were obtained after data cleaning. The demographic characteristics, such as gender, age, educational level, religion, ethnicity, and EDC type, are detailed in Table 1.

### 2.2. Measures

The instruments were translated and culturally adapted for Burmese participants in accordance with the VIA Institute’s translation guidelines (Bates & McGrath, 2018). The expert panel comprised four specialists. Two bilingual translators, one with expertise in psychological constructs and the other an English language teacher, independently translated the questionnaires. A third translator reconciled these two versions into a single Burmese version, and a fourth translator conducted a back translation. The expert panel and the first author finalized the Burmese version to ensure semantic, idiomatic, and conceptual equivalence. Pre-testing was conducted with ten student teachers from the targeted population to assess linguistic clarity. Following minor corrections, the Burmese version of the questionnaires was finalized. An independent pilot testing (*n* = 304) with student teachers from five EDCs was conducted to inform the conceptual basis of the variables in the present analysis. The findings of the exploratory factor analysis (EFA) revealed a parsimonious four-factor structure for VIA-72: intellectual and emotional, intrapersonal, interpersonal, and civic strengths; a unidimensional structure for strengths self-efficacy, and flourishing. For resilience, we used the adapted Burmese version of CD-RISC-25 (Phyu & Han, 2021). All measures revealed a good reliability, with an average of 0.80.

#### 2.2.1. Burmese Version of VIA-72

Twenty-four character strengths were assessed using a short version of the Values in Action Inventory of Strengths-72 (VIA-72; Peterson & Seligman, 2004) with a 5-point Likert scale ranging from 1 = very much unlike me to 5 = very much like me. The scale had 72 items divided into four dimensions: intellectual and emotional, intrapersonal, interpersonal, and civic strengths. For each participant, mean scores were computed for the four higher-order dimensions by averaging the relevant strengths, with higher values indicating a greater endorsement of the respective strengths domain. The confirmatory factor analysis (CFA; *n* = 1251) of the Burmese VIA-72 supported the four-factor model identified in the pilot test. (χ^2^ (chi-square)/*df* (degrees of freedom) = 5.852, CFI (comparative fit index) = 0.891, TLI (Tucker–Lewis index) = 0.877, RMSEA (root mean square error) = 0.060, 90% CI [0.057, 0.063], SRMR (standardized root mean-square) = 0.018). The reliability values for the four-factor structure of the Burmese version were satisfactory (Cronbach’s α = 0.64–0.80; McDonald’s ω = 0.64–0.80).

#### 2.2.2. Burmese Version of the Strengths Self-Efficacy Scale (SSES)

The SSES (11-item) was used to evaluate the confidence of student teachers in applying their personal strengths to achieve the targeted goals and a successful life. The scale was developed by Tsai et al. (2014) to help individuals recognize their strengths and reflect on the application of their strengths in daily life. Each item is scored on an 11-point scale (0 = not confident at all, 5 = moderately confident, and 10 = extremely confident). The mean score for strengths self-efficacy was calculated by averaging all 11 items, with higher scores indicating stronger perceived efficacy in applying personal strengths. In the present study, CFA (*n* = 1251) for the Burmese version of SSES showed an adequate unidimensional model fit (χ^2^/*df* = 6.67, CFI = 0.969, TLI = 0.957, RMSEA = 0.067, 90% CI [0.060, 0.075]) with high internal consistency (Cronbach’s α = 0.915; McDonald’s ω = 0.916).

#### 2.2.3. Connor–Davidson Resilience Scale 25 (CD-RISC-25)

Resilience was measured with the Burmese version of CD-RISC-25 to assess an individual’s ability to adapt and recover from adversity (Davidson & Connor, 2018). The scale consists of 25 items, and the student teachers rated on a 5-point scale ranging from 0 = not true at all to 4 = true nearly all the time. The total resilience score was computed by averaging the 25 items, with higher mean scores reflecting greater resilience. CFA (*n* = 1251) in the current sample demonstrated an acceptable one-factor structure (χ^2^/*df* = 6.191, CFI = 0.851, TLI = 0.835, RMSEA = 0.064, 90% CI [0.061, 0.067], SRMR = 0.048) and high reliability (Cronbach’s α = 0.897; McDonald’s ω = 0.898).

#### 2.2.4. Flourishing Scale (FS)

Different aspects of psychological well-being were measured using FS (Diener et al., 2010). It contains 8 items to assess the purpose of life, positive relationships, commitment, competence, self-esteem, optimism, and contribution to others’ well-being. The responses are given on a 7-point Likert scale (1 = strongly disagree to 7 = strongly agree). A total flourishing score was calculated by averaging the 8 items, with higher scores representing greater flourishing. The Burmese version of FS confirmed the unidimensional structure in CFA (*n* = 1251) with fit indices (χ^2^/*df* = 5.392, CFI = 0.971, TLI = 0.955, RMSEA = 0.059, 90% CI [0.048, 0.071], SRMR = 0.038), supporting the adequacy of the single-factor structure of the Burmese FS with good internal consistency (Cronbach’s α = 0.824; McDonald’s ω = 0.824).

### 2.3. Data Analysis

The study aimed to conduct a parallel mediational analysis to investigate the indirect effects of character strengths (independent variable) on flourishing (dependent variable) among Myanmar student teachers through strengths self-efficacy and resilience as mediators. Parallel mediation helps cultivate a better understanding of the complex relationships between the variables, highlighting the most significant mediator (Rucker et al., 2011). The statistical analyses were conducted using JASP version 0.95.1.0 (JASP Team, 2018), IBM SPSS Statistics 23 (IBM Corp., 2015b), and AMOS 23 (IBM Corp., 2015a). Analyses were performed in three steps: (a) preliminary analysis, (b) measurement model evaluation through CFA, and (c) structural equation modeling (SEM).

(a) Preliminary analysis. First, data were screened for missing values and accuracy. Then, standardized z-scores were examined to assess univariate outliers (z ± 3.29), while Mahalanobis distance (*p* < 0.001) was used to detect multivariate outliers (Tabachnick et al., 2019). Twenty-six cases were found to be potential multivariate outliers. Inspecting data entry errors, identical responses, and impossible values indicated valid response patterns, and all cases were retained. Bootstrap estimation was used to ensure robustness. Accordingly, univariate normality is checked for the variables intellectual and emotional (CS 1), intrapersonal (CS 2), interpersonal (CS 3), civic (CS 4), character strengths (CSs), strengths self-efficacy (SSE), resilience (R), and flourishing (F). The distribution of each variable fell within the acceptable cut-off values for normality (skewness ≤ 2 and kurtosis ≤ 7) (Kline, 2015). Multicollinearity was examined through the variance inflation factor (VIF < 5.0), and tolerance values > 0.20 demonstrate satisfactory independence of predictors (Hair et al., 2021). The results of the present study indicated that multicollinearity was not a concern. The conventional cut-off values for internal consistency (Cronbach’s alpha: α and McDonald’s omega: ω) were used in accordance with the criteria suggested by Kline (2015), indicating excellent (α > 0.90), good (α = 0.89–0.80), and acceptable (α = 0.79–0.70) reliability. Moreover, we assessed the composite reliability (CR > 0.70), average variance extracted (AVE > 0.50), and maximum shared variance (MSV) to ensure convergent and discriminant validity of the study variables. Pearson’s correlations were computed to provide preliminary evidence of linear associations between variables before SEM testing. The correlation coefficients are classified as follows: 0.90–1.00 = very strong, 0.70–0.89 = strong, 0.40–0.69 = moderate, 0.10–0.39 = weak, and 0.00–0.10 = negligible.

(b) Measurement model evaluation through CFA. Prior to the structural model, confirmatory factor analysis of the full measurement model was conducted to ensure psychometric adequacy. We followed Hu and Bentler’s (1999) recommendations for good conventional goodness-of-fit indices: chi-square/degrees of freedom (CMIN/DF ≤ 3), comparative fit index and Tucker–Lewis index (CFI and TLI ≥ 0.95), root mean square error (RMSEA ≤ 0.06), and standardized root mean-square (SRMR ≤ 0.08), incremental fit index (IFI > 0.95), goodness of fit index and adjusted goodness of fit (GFI and AGFI > 0.95), normed fit index and relative fit index (NFI and RFI > 0.95). The four dimensions of character strengths (CS): intellectual and emotional (CS 1), intrapersonal (CS 2), interpersonal (CS 3), and civic strengths (CS 4) were treated as indicators of CSs. The unidimensional constructs (strengths self-efficacy, resilience, and flourishing) were represented as balanced parcels created on standardized factor loadings identified during the CFA. Each parcel contains items with low, medium, and high loadings to ensure balanced indicators and stable measurement properties of the constructs. We adopted this approach to reinforce the stable factor structures identified in the EFA conducted with an independent pilot sample. Parcel-level CFAs also confirmed satisfactory model fits for each scale.

(c) Structural equation modeling (SEM). The hypothesized parallel mediation model was evaluated through SEM. Character strengths (CSs), strengths self-efficacy (SSE), resilience (R), and flourishing (F) were treated as latent variables. CS 1 to 4 were represented as indicators of CSs, three parcels for SSE and F, and four parcels for R. The structural paths were estimated using maximum likelihood, and indirect effects were tested via bootstrapping procedures based on 5000 resamples with bias-corrected 95% confidence intervals.

## 3. Results

### 3.1. Preliminary Analysis

#### 3.1.1. Descriptive Statistics

The distributional properties of the study variables were examined before conducting correlational and mediational analyses. As presented in Table 2, the skewness ranged from −1.61 to 0.31, and kurtosis ranged from 0.04 to 5.25, indicating that these thresholds were within the acceptable criteria for appropriate normality (skewness ≤ 2 and kurtosis ≤ 7; Kline, 2015). The descriptive statistics demonstrated that student teachers reported moderate endorsement of character strengths across four domains, strong confidence in using personal strengths, moderate resilience, and high levels of flourishing.

#### 3.1.2. Multicollinearity Diagnostics

Multicollinearity among the predictor variables was evaluated and found to be absent in the current sample. Gender was incorporated as a control variable in the structural analysis, as prior research indicated significant differences, and contextual factors may influence the study variables. EDC type and age were excluded as they are unlikely to introduce substantial differences in the variables. Each predictor variable demonstrated acceptable values (VIF < 5.0; tolerance > 0.20; Hair et al., 2021): VIFs ranged from 1.056 to 1.511, and the tolerance ranged from 0.662 to 0.947. These results (see Table 3) indicated that the variables included in the structural models were free from multicollinearity concerns.

#### 3.1.3. Reliability and Validity

The internal consistency was evaluated using Cronbach’s alpha (α) and composite reliability (CR). As indicated in Table 4, the reliability values for each construct were acceptable, with CR values exceeding the recommended threshold of 0.70. Although the Cronbach’s alpha value for character strengths was slightly below the conventional cut-off point, the composite reliability indicated satisfactory internal consistency. To assess convergent and discriminant validity, we calculated the average variance extracted (AVE) and maximum shared variance (MSV). The findings revealed that AVE values, ranging from 0.66 to 0.80, indicated good convergent validity. Discriminant validity was confirmed by ensuring that AVE values exceeded the MSV for all constructs (Fornell & Larcker, 1981; Hair et al., 2010). These validity indices were derived from the standardized factor loadings obtained from the confirmatory factor analysis of the full measurement model. Overall, the measurement model demonstrates satisfactory reliability, convergent validity, and discriminant validity.

#### 3.1.4. Pearson Correlation Among the Study Variables

Pearson correlation analyses were conducted to investigate the relationships between variables before structural equation modeling (see Table 5). As expected, character strengths (CSs) and their four dimensions were positively and significantly related to SSE (*r* = 0.183–0.224, *p* < 0.01), resilience (*r* = 0.343–0.432, *p* < 0.01), and flourishing (*r* = 0.248–0.358, *p* < 0.01). SSE demonstrated a moderate positive correlation with resilience (*r* = 0.446, *p* < 0.01) and flourishing (*r* = 0.391, *p* < 0.01). In addition, resilience was also moderately correlated with flourishing (*r* = 0.587, *p* < 0.01).

Regarding demographic variables, gender was weakly related to CSs dimensions (*r* = 0.099–0.268, *p* < 0.01) and was not significantly related to SSE, resilience, and flourishing. Age and the type of EDC showed very small or negligible associations with the focal constructs. Specifically, age was weakly correlated with CS 1 (*r* = 0.089, *p* < 0.01) and the type of EDC (*r* = −0.118, *p* < 0.01). The EDC type showed weak relationships with CS 1 (*r* = −0.161, *p* < 0.01) and CSs (*r* = −0.075, *p* < 0.01). Generally, the correlation among the study variables supported the hypothesized mediational model, indicating a significant associations among the constructs. The correlation coefficients remained below 0.90, indicating that there was no risk of multicollinearity at the bivariate level (Kline, 2015).

The small but statistically significant associations between gender and character strengths supported its inclusion as a control variable in the structural model. Age and type of EDC were excluded due to their non-significant and negligible associations with the study variables, as well as the restricted age range of the sample. Preliminary analyses indicated that controlling for age and type of EDC did not meaningfully alter the correlation patterns, suggesting minimal confounding influence. Thus, these demographic variables were not controlled in the following analyses. Gender, however, has been incorporated as a control variable, given its potential influence on the relationships among the study variables (Casali et al., 2021; Martínez-Martí & Ruch, 2017).

#### 3.1.5. Measurement Model Evaluation

The full measurement model was analyzed using confirmatory factor analysis (CFA) to assess the adequate model fit. It consisted of four latent variables: Character strengths (CSs), strengths self-efficacy (SSE), resilience, and flourishing. CSs was represented by four dimensions: intellectual and emotional (CS 1), intrapersonal (CS 2), interpersonal (CS 3), and civic strengths (CS 4). For the unidimensional measures: SSE, resilience, and flourishing, parcels were formed to reduce inflated measurement errors and achieve parsimonious structural estimation. The parceling procedure was based on the standardized factor loadings obtained from CFA conducted with the current study sample (*n* = 1251), following the item-to-construct balance approach (Little et al., 2002). For SSE, three parcels were created: SSE_P1 (items 5, 8, and 2), SSE_P2 (items 10, 9, and 1), and SSE_P3 (items 7, 6, 4, 11, and 3). Resilience was represented by four parcels: R_P1 (items 17, 7, 20, 13, 3, and 2), R_P2 (items 11, 5, 10, 19, 6, and 1), R_P3 (items 16, 15, 9, 25, 23, and 18), and R_P4 (items 24, 12, 14, 4, 22, 21, and 8). Flourishing was modeled using three parcels: F_P1 (items 5, 7, and 2), F_P2 (items 3, 8, and 1), and F_P3 (items 4 and 6).

We computed multiple indices, including the chi-square to degrees of freedom ratio (CMIN/DF), Goodness-of-Fit Index (GFI), Adjusted Goodness-of-Fit Index (AGFI), Normed Fit Index (NFI), Relative Fit Index (RFI), Incremental Fit Index (IFI), Comparative Fit Index (CFI), Tucker–Lewis Index (TLI), Root Mean Square Error of Approximation (RMSEA), and Standardized Root Mean Square Residual (SRMR). The recommendations of Hu and Bentler (1999) and Kline (2015) were followed to determine the goodness of fit of the measurement and structural models. Given the larger sample size, the model evaluation relied on incremental and residual-based indices. The measurement model demonstrated an overall acceptable fit to the data: χ^2^ (82, N = 1251) = 567.030 (*p* < 0.001), CMIN/DF = 6.915, GFI = 0.945, AGFI = 0.919, NFI = 0.950, RFI = 0.936, IFI = 0.957, CFI = 0.957, TLI = 0.945, RMSEA = 0.069, and SRMR = 0.117. Most incremental and absolute fit indices (GFI, AGFI, NFI, RFI, IFI, CFI, and TLI) exceeded the recommended threshold of 0.90, indicating good model fit. The RMSEA value indicated an acceptable fit (≤0.08). Although the CMIN/DF value exceeded the recommended cut-off point, this inflation was commonly observed in larger samples. However, the SRMR value was higher than the recommended threshold of 0.08, indicating some degree of misfit within the model. Nevertheless, when evaluating the majority of fit indices alongside the theoretical consistency of the model, it is concluded that the measurement model remains acceptable for continuation in subsequent analyses.

#### 3.1.6. Structural Model Evaluation

**Figure 2:**
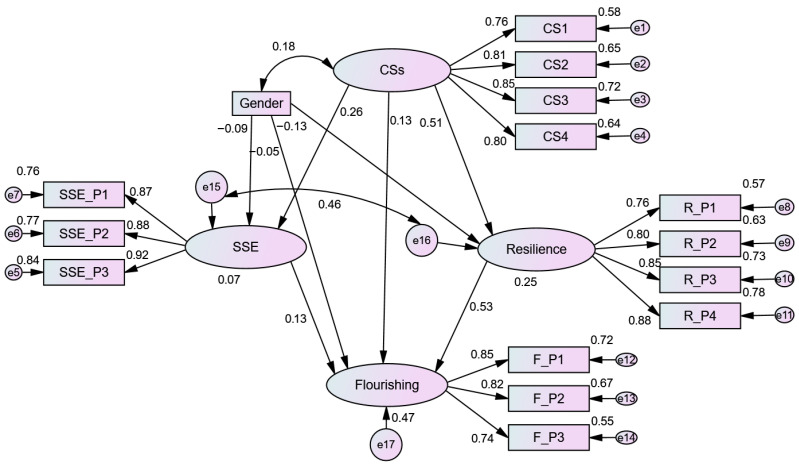
A parallel mediation model examining the direct and indirect effects of character strengths on flourishing through strengths self-efficacy and resilience. CSs = character strengths; CS 1 = intellectual and emotional strengths; CS 2 = intrapersonal strengths; CS 3 = interpersonal strengths; CS 4 = civic strengths; SSE = strengths self-efficacy; P = parcel.

Following measurement model validation, structural equation modeling (SEM) with maximum likelihood estimation was used to test a hypothesized parallel mediation model, positing that character strengths predicted flourishing directly and indirectly through strengths self-efficacy and resilience. The structural model showed a good overall fit [χ^2^ (81, N = 1251) = 346.610 (*p* < 0.001), CMIN/DF = 4.279, GFI = 0.965, AGFI = 0.948, NFI = 0.969, RFI = 0.960, IFI = 0.976, CFI = 0.976, TLI = 0.969, RMSEA = 0.051, and SRMR = 0.018]. Gender was included as a covariate to predict SSE, resilience, and flourishing. Figure 2 shows the standardized estimates among the variables.

### 3.2. Direct Effects

The results (see Table 6) indicated that the character strengths significantly predicted SSE (β = 0.261, *p* < 0.001) and resilience (β = 0.505, *p* < 0.001). All reported path coefficients are standardized estimates. The SSE mediator was found to positively predict flourishing (β = 0.130, *p* < 0.001), and resilience also emerged as a strong predictor of flourishing (β = 0.535, *p* < 0.001). Importantly, the direct effect of character strengths on flourishing remained (β = 0.134, *p* < 0.001), indicating a partial mediation. Regarding the control variable, gender showed significant but weak negative effects on strengths self-efficacy (β = −0.088, *p* < 0.01) and resilience (β = −0.134, *p* < 0.001), suggesting that male student teachers reported slightly lower levels of confidence in applying their personal strengths and adaptive capacity compared to female student teachers. However, the magnitude of these effects was small and of limited practical significance. Gender did not have a significant direct effect on flourishing (β = −0.047, *p* > 0.05). Overall, the inclusion of gender as a control variable for potential cofounding influences did not influence the structural relationships among the focal constructs (Williams et al., 2009). These findings align with the previous study, which demonstrated that gender did not differentially impact resilience and self-efficacy among preservice teachers in Kazakhstan (Shuakbayeva et al., 2025).

### 3.3. Indirect and Mediation Effects

The mediational roles of strengths self-efficacy and resilience were tested using a bias-corrected bootstrap procedure with 95% confidence intervals. Indirect effects were estimated using user-defined estimands in AMOS. Although statistical significance was evaluated based on unstandardized estimates, standardized coefficients (β) are also reported for ease of interpretation. The results indicated that character strengths had significant indirect effects on flourishing through both mediators. The standardized indirect effect through resilience (β = 0.270, *p* < 0.001) was stronger than that through strengths self-efficacy (β = 0.034, *p* < 0.001), indicating that resilience serves as a more significant mediator in predicting flourishing among student teachers in Myanmar. Consistent with these findings, bootstrapped unstandardized indirect effects were also significant (see Table 7), with confidence intervals that did not include zero. These results support the presence of parallel mediation, whereby both strengths self-efficacy and resilience significantly transmit the effect of character strengths on flourishing (De la Fuente et al., 2022; Zhao et al., 2021). Furthermore, the model explained 7.3% of the variance in strengths self-efficacy, 24.9% in resilience, and 47.3% in flourishing, indicating substantial explanatory power, particularly for flourishing.

## 4. Discussion

This study aimed to investigate the role of character strengths in predicting flourishing among Myanmar student teachers through strengths self-efficacy (SSE) and resilience as mediators. The study proposed complementary psychological pathways and extended previous research that has assessed these mediators in isolation (Botha et al., 2022; Collins, 2009; De la Fuente et al., 2022).

Regarding the relationships between the constructs (see Table 5), the findings were consistent with previous research: student teachers’ character strengths were positively related to SSE (Casali et al., 2021; Collins, 2009), resilience (Chung, 2008), and flourishing (Wagner et al., 2021). The association of character strengths with resilience and flourishing revealed stronger associations than SSE, suggesting that character strengths may be more directly related to adaptive coping and functioning effectively than to efficacy beliefs about the use of strengths. At the dimensional level, all four strengths were positively related to SSE, resilience, and flourishing, with intrapersonal and interpersonal strengths showing comparatively stronger relationships with resilience and flourishing. This pattern suggests that student teachers who demonstrate a high prevalence of interpersonal and intrapersonal strengths scored highly on resilience and flourishing. Furthermore, as compared to SSE, resilience had the strongest association with flourishing (Yildirim & Belen, 2019).

Generally, the demographic variables (age and EDC type) showed negligible associations with study variables. Consistent with the preliminary and main analyses, these variables did not demonstrate meaningful relationships with the core constructs and were therefore not retained in the final model. Gender differences have been found, and this weak association may serve as a cofounder in mediational analysis. It is important to account for the theoretically relevant covariates when investigating direct and indirect effects (Becker, 2005; Kline, 2015). Consequently, gender was included as a control variable in the SEM model to strengthen internal validity and improve the precision of the mediation estimates. Although statistically significant, the effect of gender was minimal, indicating that it does not greatly influence the relationships between character strengths, SSE, resilience, and flourishing. Its inclusion primarily serves to enhance the robustness of the estimated pathways. This finding is consistent with previous literature suggesting that gender differences in these constructs are generally modest and context-dependent, reinforcing the perspective that the proposed psychological mechanisms function similarly across gender groups (Shuakbayeva et al., 2025; Zhao et al., 2021).

The confirmatory factor analyses of the measurement and structural models confirmed the applicability of the proposed parallel mediation model. In addition, the measurement model demonstrated satisfactory reliability and validity, providing support for the adequacy of the constructs used in the subsequent structural analysis. Using structural equation modeling, it is concluded that character strengths positively predicted the flourishing of student teachers both directly and indirectly. Importantly, resilience emerged as a more dominant predictor compared to SSE within the context of Teacher Education, indicating the crucial role of resilience in fostering the flourishing of student teachers. The SEM results of the current study supported Hypothesis 1, providing evidence that character strengths had direct effects on SSE, resilience, and flourishing. From a theoretical point of view, this supports the view that character strengths operate as psychological resources that underpin the confidence of student teachers in applying their strengths, the ability to bounce back from adversity, and flourishing. Consistent with the previous literature, character strengths have been shown to explain significant variance in resilience beyond related psychological constructs (Martínez-Martí & Ruch, 2017), to be important positive personality traits for high self-efficacy beliefs (Weber et al., 2013), and to serve as a strong predictor of flourishing (Ekşi et al., 2022).

Hypothesis 2 confirmed the indirect effects of character strengths on flourishing through SSE and resilience. The standardized estimates indicated that SSE significantly mediated the relationship between character strengths and flourishing (β = 0.034), while resilience contributed more to the association (β = 0.270). Importantly, the direct effect of character strengths on flourishing remained significant after including both mediators (β = 0.134), indicating a partial mediation. The study explored additional mechanisms to better explain the association between character strengths and flourishing by integrating a conceptually relevant network with resilience and SSE in the Teacher Education context. This pattern is theoretically coherent: individuals who believe in their ability to use personal strengths are more likely to engage in constructive activities that support improved well-being (Bandura, 1997; Tsai et al., 2014); however, the magnitude of the effect size is insufficient to fully explain the link between character strengths and flourishing.

In light of the unstable and tough political climate since 2021 and its repercussions on education, the economy, and the challenges of meeting basic needs, resilience has emerged as an immediate and pragmatic regulatory mechanism (Win, 2024). It enables student teachers to navigate ongoing academic and contextual stressors. In contrast, SSE represents a belief-based motivational process that may exert its influence more indirectly through behavior. The study’s findings reflect the context of Myanmar Teacher Education, where social and educational uncertainties can increase the importance of resilience in maintaining flourishing. Furthermore, student teachers are expected to perform well in teaching, research training, and practicum experience. In such context, the role of resilience becomes essential in facing societal demands with a broader adaptive capacity, including emotional regulation, flexibility, and recovery from adversity, thereby serving as a proximal determinant for flourishing (Davidson & Connor, 2018). Reasonably, character strengths contribute to flourishing by fortifying the ability of student teachers to withstand and adapt to stressors, rather than solely by increasing the confidence in strength use. These findings highlight a dual-path mechanism in which SSE functions as a motivational pathway, whereas resilience operates as an adaptive-regulatory pathway with more immediate implications for flourishing. This is theoretically relevant and should be emphasized in the preparation of future intervention studies in the Teacher Education context.

Furthermore, the present findings about the mediating effect of resilience are stronger than previous research (De la Fuente et al., 2022), which identified resilience as a single mediator of the relationship between character strengths and flourishing among Spanish university students (β = 0.219). Wang and Ding (2023) provided further evidence that SSE served as a significant mediator in the association between character strengths-based leadership and strengths use in the Chinese workplace (β = 0.102). The structural model of this study explained 47.3% of the variance in flourishing. Specifically, character strengths accounted for meaningful variance in resilience (24.9%) and a smaller but significant variance in SSE (7.3%). Combined, these findings demonstrate that character strengths are meaningful predictors of flourishing in this population. This sheds light on the important implications for Teacher Education: first, resilience-oriented programs may represent a promising avenue for strengths-based interventions to enhance well-being and flourishing; second, in an applied context, SSE may serve as a useful indicator to assess the participants’ confidence level; third, the academic interventions that prioritize character strengths are aligned with the competency-based curriculum (Lavy, 2020; Mar et al., 2025). Therefore, it is worth considering integrating strengths-based activities and resilience-supportive strategies in the teaching–learning environment.

According to the findings of this study, some limitations and directions for future research should also be noted. First, the application of a cross-sectional research design precludes causal inference. The structural model was based on the theoretical foundation and showed a good fit; however, the temporal ordering of character strengths, SSE, resilience, and flourishing could not be conclusively established. It remains plausible, for example, that flourishing reinforces resilience or strengthens efficacy beliefs over time. In order to clarify directionality and examine potential reciprocal relationships among these constructs, longitudinal or experimental designs are recommended. Second, VIA-72 is a short measure of VIA-IS, and its use of high-order factor structure maximizes parsimony and aligns with theoretical conceptualizations of strengths. However, it limits the insights into the relative contribution of specific character strengths domains (e.g., interpersonal vs. intrapersonal strengths). As resilience emerged as the dominant mediator, future studies should examine whether particular strengths are differentially predictive. Third, while resilience demonstrated a stronger mediating effect than SSE, it is unclear whether these mechanisms work independently or sequentially over time. Future studies could explore serial mediation models (e.g., character strengths → SSE → resilience → flourishing) to determine whether efficacy beliefs enhance adaptive capacity, which in turn enhances flourishing. Finally, the findings of the study are based on the Myanmar Teacher Education context and may not be generalizable to other countries’ contexts. Cross-cultural replication studies are recommended to improve the generalizability of the proposed mediational model and to further clarify the link between character strengths and flourishing.

## 5. Conclusions

To sum up, the study provides empirical evidence that the character strengths are significant predictors of flourishing among student teachers, with resilience as a strong mediator and strengths self-efficacy (SSE) as a supporting mechanism. This finding expands the literature by illustrating that flourishing is not merely the result of possessing personal strengths; it also encompasses the ability to adapt to challenges by effectively mobilizing these strengths in everyday life. Achieving a state of flourishing can be particularly difficult within a challenging educational environment. Hence, student teachers must enhance their resilience when applying character strengths in order to truly flourish, indicating that resilience is more vital than self-efficacy beliefs in sustaining flourishing. This distinction deepens the current understanding by clarifying that merely believing in one’s strengths is insufficient without the capacity to cope with and recover from stressors. From a broader perspective, the study highlights the importance of integrating both motivational (SSE) and adaptive (resilience) processes in promoting flourishing, particularly in contexts characterized by uncertainty and high demands, such as Teacher Education (Kutsyuruba et al., 2019). Ultimately, the study underscores that fostering flourishing in Teacher Education requires not only the cultivation of strengths but also the systematic development of resilience necessary to effectively utilize these strengths in complex and demanding environments.

## Figures and Tables

**Table 1:** Demographic characteristics of student teachers (*n* = 1251).

Variable	Category	*n*	%
Gender	Female	851	68.0
	Male	400	32.0
Age	18–20 years	524	41.9
	21–25 years	727	58.1
Type of EDC	Upper Myanmar EDC 1	319	25.5
	Upper Myanmar EDC 2	307	24.5
	Lower Myanmar EDC 3	350	28.0
	Lower Myanmar EDC 4	275	22.0
Educational level	Year 1	174	13.9
	Year 2	119	9.5
	Year 3	537	42.9
	Year 4	421	33.7
Religion	Buddhist	1226	98.0
	Christian	21	1.7
	Islam	4	0.3
Ethnicity	Bamar	1118	89.4
	Mixed ethnicity	41	3.3
	Others (Chin, Shan, Rakhine, Kayin)	92	7.4

Note. EDC = Education Degree College.

**Table 2:** Descriptive statistics of the study variables.

Variables	Minimum	Maximum	*M*	SD	Skewness	Kurtosis
Intellectual and emotional	2.22	5.00	3.79	0.44	0.10	0.23
Intrapersonal	2.43	5.00	3.98	0.38	−0.05	0.62
Interpersonal	2.62	5.00	3.96	0.41	0.04	0.17
Civic	2.17	5.00	3.94	0.40	0.02	0.63
Character strengths	2.58	5.00	3.92	0.35	0.31	0.68
Strengths self-efficacy	0.91	10.00	6.25	1.86	−0.43	0.04
Resilience	0.92	4.00	2.90	0.52	−0.39	0.25
Flourishing	1.00	7.00	5.70	0.74	−1.61	5.25

**Table 3:** Multicollinearity statistics on student teachers’ flourishing.

Predictor	Beta	*t*	Sig.	VIF	Tolerance
Character strengths	0.124	4.99	0.000	1.230	0.813
Strengths self-efficacy	0.152	6.013	0.000	1.287	0.777
Resilience	0.460	16.796	0.000	1.511	0.662
Gender	−0.046	−2.019	0.044	1.056	0.947

**Table 4:** Reliability and validity of the study variables.

Variable	α	CR	AVE	MSV
Character strengths	0.68	0.88	0.65	0.19
Strengths self-efficacy	0.915	0.92	0.80	0.22
Resilience	0.897	0.90	0.70	0.35
Flourishing	0.824	0.85	0.66	0.34

Note. AVE = average variance extracted; CR = composite reliability; MSV = maximum shared variance; α = Cronbach’s alpha.

**Table 5:** Pearson correlations among the socio-demographic factors and the study variables.

Variables		1	2	3	4	5	6	7	8	9	10	11
1	Gender	1										
2	Age	0.360 **	1									
3	EDC Type	0.016	−0.118 **	1								
	Study											
4	Intellectual and emotional	0.268 **	0.089 **	−0.161 **	1							
5	Intrapersonal	0.118 **	0.027	−0.065 *	0.626 **	1						
6	Interpersonal	0.121 **	−0.018	−0.018	0.665 **	0.652 **	1					
7	Civic	0.099 **	−0.001	−0.003	0.558 **	0.672 **	0.697 **	1				
8	Character strengths	0.181 **	0.030	−0.075 **	0.841 **	0.854 **	0.880 **	0.850 **	1			
9	Strengths self-efficacy	−0.040	0.012	−0.029	0.211 **	0.184 **	0.189 **	0.183 **	0.224 **	1		
10	Resilience	−0.046	−0.046	−0.015	0.348 **	0.400 **	0.390 **	0.343 **	0.432 **	0.466 **	1	
11	Flourishing	−0.048	−0.005	0.006	0.248 **	0.333 **	0.333 **	0.318 **	0.358 **	0.391 **	0.587 **	1

Note. * *p* < 0.05; ** *p* < 0.01.

**Table 6:** Direct effects of the study variables (95-biased corrected confidence intervals based on 5000 resamples; *n* = 1251).

	Standardized Estimates		
	Beta	CI (95%)	Sig.
Direct effects			
CSs → SSE (a1)	0.261	[0.193, 0.330]	<0.001
CSs → Resilience (a2)	0.505	[0.439, 0.566]	<0.001
SSE → Flourishing (b1)	0.130	[0.066, 0.194]	<0.001
Resilience → Flourishing (b2)	0.535	[0.448, 0.618]	<0.001
CSs → Flourishing (c′)	0.134	[0.058, 0.211]	0.001

Note. CI = confidence interval.

**Table 7:** Bootstrapping unstandardized indirect effects predicting flourishing (95% bias-corrected confidence intervals based on 5000 resamples; *n* = 1251).

Relationship	B	*SE*	*t*	CI (95%)	Sig.
CSs → SSE → Flourishing	0.070	0.020	3.5	[0.036, 0.115]	<0.001
CSs → Resilience → Flourishing	0.559	0.061	9.164	[0.452, 0.692]	<0.001

Note. B = unstandardized estimate; CI = confidence interval; SE = standard error. Indirect effects were estimated using user-defined estimands in AMOS based on unstandardized coefficients.

## Data Availability

The data supporting the findings of this study are available in the Open Science Framework (OSF) repository at: https://osf.io/84g2x/overview?view_only=690f00b3e4184a8cb7f3077f1138279f (accessed on 13 March 2026).
